# Placental ischemia increases seizure susceptibility and cerebrospinal fluid cytokines

**DOI:** 10.14814/phy2.12634

**Published:** 2015-11-24

**Authors:** Junie P Warrington

**Affiliations:** Department of Physiology & Biophysics, University of Mississippi Medical CenterJackson, Mississippi

**Keywords:** Cerebrospinal fluid, cytokine, eclampsia, neurokinin B, placental ischemia, seizure

## Abstract

Eclampsia is diagnosed in preeclamptic patients who develop unexplained seizures and/or coma during pregnancy or postpartum. Eclampsia is one of the leading causes of maternal and infant morbidity and mortality, accounting for ∼13% of maternal deaths worldwide. Little is known about the mechanisms contributing to the pathophysiology of eclampsia, partly due to the lack of suitable animal models. This study tested the hypothesis that placental ischemia, induced by reducing utero-placental perfusion, increases susceptibility to seizures, cerebrospinal fluid (CSF) inflammation, and neurokinin B (NKB) expression in brain and plasma. Pentylenetetrazol (PTZ), a pro-convulsive drug, was injected into pregnant and placental ischemic rats (40 mg/kg, i.p.) on gestational day 19 followed by video monitoring for 30 min. Seizure scoring was blindly conducted. Placental ischemia hastened the onset of seizures compared to pregnant controls but had no effect on seizure duration. Placental ischemia increased CSF levels of IL-2, IL-17, IL-18 and eotaxin (CCL11), had no effect on plasma NKB; however, PTZ increased plasma NKB in both pregnant and placental ischemic rats. NKB was strongly correlated with latency to seizure in normal pregnant rats (*R*^2^ = 0.88 vs. 0.02 in placental ischemic rats). Lastly, NKB decreased in the anterior cerebrum in response to placental ischemia and PTZ treatment but was unchanged in the posterior cerebrum. These data demonstrate that placental ischemia is associated with increased susceptibility to seizures and CSF inflammation; thus provides an excellent model for elucidating mechanisms of eclampsia-like symptoms. Further studies are required to determine the role of CSF cytokines/chemokines in mediating increased seizure susceptibility.

## Introduction

Preeclampsia is a pregnancy disorder characterized by new-onset hypertension after the 20th week of gestation, accompanied by proteinuria or in the absence of proteinuria, low platelet count, renal insufficiency, impaired liver function, pulmonary edema, or cerebral or visual symptoms (American College of Obstetricians and Gynecologists, 2013). Preeclampsia affects 5–8% of all pregnancies in the United States (Saftlas et al. 1990) and close to 20% of African American pregnancies (Mostello et al. 2002). Currently, early delivery of the fetus and removal of the placenta are the only methods to reverse the disorder (Sibai et al. 2005; Sibai 2006), contributing to increased preterm births. Preeclampsia is a multi-organ disorder, affecting the kidney, liver, and brain. Importantly, of all preeclampsia/eclampsia-related deaths, cerebrovascular events are the cause in ∼40% of cases (MacKay et al. 2001). Additionally, women with preeclampsia present with neurological symptoms such as headaches, blurred vision, nausea, drowsiness, and seizures (in the case of eclampsia) (Chakravarty and Chakrabarti 2002). Furthermore, women with preeclampsia have increased risk of stroke during pregnancy and postpartum (Tang et al. 2009).

Preeclampsia can quickly progress to eclampsia, diagnosed in preeclampsia patients without a history of epilepsy, presenting with new onset seizures or convulsions during pregnancy or postpartum. According to the Preeclampsia Foundation, eclampsia is one of the top five causes of maternal and infant mortality and morbidity and is responsible for approximately 13% of all maternal deaths worldwide (Nour 2008). Importantly, eclampsia has been described as the highest predictor of postpartum acute cerebrovascular disease (Hovsepian et al. 2014). Despite these statistics, the mechanisms contributing to the onset of eclampsia symptoms are not clear due to immediate intervention in the form of delivery following stabilization in women with eclamptic episodes as well as lack of suitable animal models.

Studies describing potential animal models of eclampsia have been recently described in which animal models of preeclampsia are used to explore susceptibility to drug-induced seizures. For example, one study induced preeclampsia-like symptoms by administering lipopolysaccharide to pregnant rats followed by pentylenetetrazol (PTZ) treatment to induce seizures and found that lipopolysaccharide-treated animals had reduced threshold to seizures (Huang et al. 2014). Another study used placental ischemia coupled with a high cholesterol diet followed by PTZ injection and reported reduced seizure threshold and increased susceptibility to seizures in response to placental ischemia and high cholesterol diet treatment (Johnson et al. 2014). While these studies provide evidence for increased seizure susceptibility in animal models of preeclampsia, it is not known whether placental ischemia by itself can induce increased sensitivity to PTZ-induced seizures.

The placental ischemia model of preeclampsia mimics the clinical condition very well. For example, like preeclamptic patients, placental ischemic rats have increased arterial blood pressure, with or without proteinuria (Alexander et al. 2001), increased inflammatory cytokines (LaMarca et al. 2005) (Gadonski et al. 2006), and increased anti-angiogenic factors (Gilbert et al. 2009) (Gilbert et al. 2007). Additionally, placental ischemic rats have impaired cerebral blood flow autoregulation and increased blood-brain barrier permeability (Warrington et al. 2014) along with impaired cerebrovascular myogenic tone and edema (Ryan et al. 2011). While placental ischemia shares numerous characteristics of preeclampsia in patients, whether placental ischemia increases seizure susceptibility and whether it can be utilized as a potential animal model of eclampsia is not known.

Neurokinin B (NKB) is a neuropeptide expressed widely in the central nervous system and peripheral nervous system. It belongs to the family of neuropeptides called tachykinins that includes neurokinin A and Substance P. NKB is found in neurons and immune cells in the nervous system but have also been shown to be expressed in placenta (Page and Lowry 2000; Page et al. 2000) in the outer syncytiotrophoblasts near the maternal circulation at term. Based on literature showing increased NKB in plasma from preeclampsia women (Page et al. 2000; D’Anna et al. 2004; Liu et al. 2009), I hypothesized that NKB will be increased in the circulation and placentas of placental ischemic rats.

This study assessed whether placental ischemic rats have increased susceptibility to pentylenetetrazol (PTZ)-induced seizures compared to normal pregnant rats. As a potential mechanism, levels of 27 cytokines/chemokines in the cerebrospinal fluid were measured. Neurokinin B (NKB) was measured in the circulation, placenta, and brain in response to placental ischemia and PTZ injection. This is the first study to assess sensitivity to seizures in response to placental ischemia and changes in cerebrospinal fluid cytokines/chemokines in an animal model of preeclampsia.

## Materials and Methods

### Animals

Timed pregnant Sprague-Dawley (CD) rats were obtained from Charles Rivers Laboratories and arrived at the Lab Animal Facilities at the University of Mississippi Medical Center on gestational day 11. All animal procedures were approved by the Institutional Animal Care and Use Committee at the University of Mississippi Medical Center. The rats were maintained on a 12 h light/12 h dark cycle and fed standard rodent chow and water ad libitum.

### Reduced uterine perfusion pressure model of placental ischemia

Placental ischemia was induced as previously described (Warrington et al. 2014). Briefly, silver clips were surgically inserted around the abdominal aorta and each of the ovarian arteries that branch from the uterine artery on day 14 of gestation (Granger et al. 2006). This method leads to a reduction of blood flow to the utero-placental unit by ∼40% in pregnant rats (Granger et al. 2006).

### Seizure induction and monitoring

On gestational day 19, normal pregnant and placental ischemic rats were injected intraperitoneally using 40 mg/kg PTZ (a sub-convulsive dose) and then placed in observation cages. Rats were then video recorded for 30 min. Seizure activity was then determined using the Racine scale by a technician in the Animal Behavior Core Facility, blinded to the treatment. Five stages of intensity were used based on the behavior of the mice: Stage 1: mouth and facial movements, Stage 2: head nodding, Stage 3: forelimb clonus, Stage 4: tonic-clonic seizures, and Stage 5: generalized tonic-clonic seizures characterized by rearing and falling. In addition to the types/stages of seizures observed, latency to first seizure and duration of seizures were also recorded.

### Collection of cerebrospinal fluid

On gestational day 19, following completion of seizure monitoring, rats were anesthetized and secured on a stereotaxic frame and cerebrospinal fluid was obtained via the cisterna magna using a butterfly needle and syringe. CSF samples were flash frozen in liquid nitrogen and stored at −80°C until processing.

### Determination of brain water content

On gestational day 19, following the collection of CSF samples, rats were euthanized under isoflurane anesthesia and brains were quickly removed and processed as described previously (Warrington et al. 2014). Briefly, the brains were hemisected along the midline and separated into anterior and posterior cerebrum. The junction of the middle cerebral artery to the circle of Willis was used as a landmark for dissection. Tissue rostral of the middle cerebral artery was designated as the anterior cerebrum and tissue caudal of the middle cerebral artery, excluding the cerebellum was considered posterior cerebrum. The brain regions were then weighed and subjected to drying in an oven at 60°C for 72 h, after which dry weight was obtained. Brain water content was then calculated as a percentage: [(wet weight − dry weight)/wet weight] × 100.

### Measurement of CSF cytokine/chemokine concentration

Cerebrospinal fluid (CSF) samples were thawed and run on the rat cytokine/chemokine magnetic bead panel (Milliplex MAP kit, Billerica, MA) which included 27 cytokines/chemokines. Manufacturer’s directions were followed and CSF samples were assayed undiluted. Cytokine/chemokine concentration was determined from a standard curve generated for each analyte.

### Assessment of Neurokinin B concentration

Neurokinin B was analyzed in the plasma, placentas, and brains of placental ischemic rats and rats were subjected to seizures using a commercially available Rat Neurokinin B EIA kit following manufacturer’s directions. Brains were collected from rats and dissected into anterior and posterior cerebrum, and flash frozen in liquid nitrogen. Anterior and posterior cerebral regions were assessed separately because previous studies demonstrate that placental ischemia preferentially affects the anterior cerebrum with no change in the posterior cerebrum (Warrington et al. 2014, 2015). Increases in brain water content and blood–brain barrier permeability were selective to the anterior cerebrum (Warrington et al. 2014). Neurokinin B concentrations were calculated using an equation generated from the standard curve as per the manufacturer’s directions.

### Statistical analysis

The significance of mean values in the normal pregnant and placental ischemic groups was determined using an unpaired *t*-test. Differences in neurokinin B concentration were determined using Two-Way ANOVA followed by Fisher’s LSD test. Comparisons were considered statistically significant if *P* < 0.05.

## Results

### Placental ischemia leads to decreased latency to seizures with no change in seizure duration

**Figure 1:**
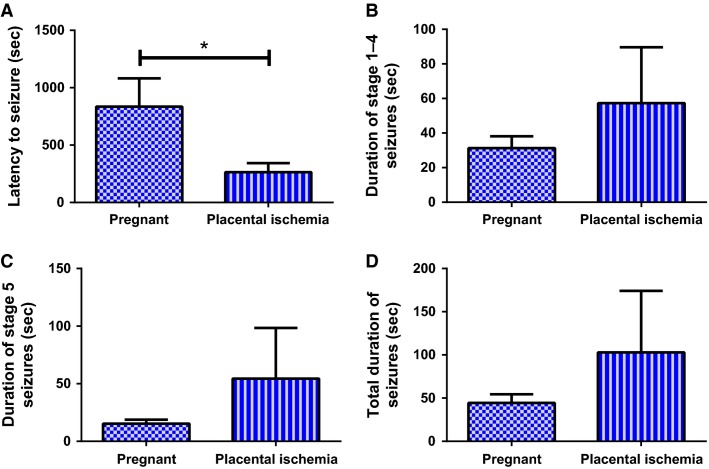
Placental ischemia increases sensitivity to PTZ-induced seizures. (A) Latency to seizure is significantly decreased in placental ischemic rats. (B) Duration of stage 1–4 seizures, (C) Duration of stage 5 seizures, and (D) Total duration of seizures tends to increase in placental ischemic group. Bars represent Mean ± SEM. *P < 0.05. *N* = 7–8 per group.

Placental ischemic rats had reduced latency to seizure (264.5 ± 78.7 sec) compared to normal pregnant rats (835.7 ± 246.6 sec; *P* < 0.05) (Fig.1A), but had a similar duration of seizures regardless of the seizure classification (*P* > 0.05). The duration of stages 1–4 seizures was 31.29 ± 6.81 sec in the normal pregnant group compared to 57.25 ± 32.38 sec in placental ischemic rats (Fig.1B, *P* = 0.238). The duration of stage 5 seizures was 15.33 ± 3.52 sec in the normal pregnant rats and 54.43 ± 43.96 sec in the placental ischemic rats (Fig.1C, *P* = 0.216). Total duration of seizures was 44.43 ± 10.06 sec in the pregnant group and 102.9 ± 71.2 sec in the placental ischemic group (Fig.1D, *P*** **= 0.231).

### Placental ischemia leads to increased cytokines/chemokines in the cerebrospinal fluid

Placental ischemia led to increased cerebrospinal fluid levels of four cytokines/chemokines (Table1, bolded) eotaxin (31.3 ± 1.4 pg/mL vs. 20.7 ± 2.9 pg/mL in pregnant rats; *P* = 0.004). Interleukin-17, a proinflammatory cytokine was also increased in the cerebrospinal fluid of placental ischemic rats (20.2 ± 3.7 pg/mL vs. 12.9 ± 1.2 pg/mL in pregnant rats; *P* = 0.034). Interleukin-18 increased from 40.1 ± 5.1 pg/mL in normal pregnant rats to 60.9 ± 8.8 pg/mL in placental ischemic rats; *P* = 0.029. Lastly, the proinflammatory cytokine, interleukin-2 was significantly increased in the placental ischemic group (59.5 ± 9.5 pg/mL) compared to the normal pregnant group (40.7 ± 4.8 pg/mL; *P* = 0.046). There was a tendency for increased levels of fractalkine (*P* = 0.069), interferon gamma (*P* = 0.057), IL-10 (*P* = 0.071), and VEGF (*P* = 0.071). The levels of all 27 cytokines/chemokines in the cerebrospinal fluid in response to placental ischemia are shown in Table1.

**Table 1:** Cerebrospinal fluid levels of cytokines/chemokines in pregnant and placental ischemic rats

Cytokine/Chemokine	Pregnant (*n* = 7)	Placental Ischemia (*n* = 6)	*P*-value
EGF (pg/mL)	ND	ND	NC
Eotaxin	**20.7 ± 2.9**	**31.3 ± 1.4**	**0.004**
Fractalkine	36.2 ± 4.2	45.0 ± 3.4	0.069
G-CSF	16.6 ± 1.7	15.6 ± 2.4	0.357
GM-CSF	184.5 ± 25.7	212.1 ± 37.0	0.272
GRO/KC/CINC	239.7 ± 30.6	243.9 ± 14.2	0.455
Interferon Gamma	42.4 ± 6.5	61.7 ± 9.6	0.057
Interleukin-10	12.4 ± 3.2	20.9 ± 4.5	0.071
Interleukin-12p70	ND	23.47* (2)	NC
Interleukin-13	4.3 ± 2.0* (5)	7.8 ± 3.7	0.200
Interleukin-1 alpha	11.2 ± 1.0* (2)	7.3 ± 2.3* (5)	0.178
Interleukin-1 beta	25.1 ± 8.4	28.7 ± 5.2	0.365
Interleukin-2	**40.7 ± 4.8**	**59.5 ± 9.5**	**0.046**
Interleukin-4	12.7 ± 2.3	16.0 ± 3.7	0.228
Interleukin-5	25.0 ± 2.9	29.8 ± 5.2	0.208
Interleukin-6	343.2 ± 58.4	367.5 ± 83.3	0.406
Interleukin-17	**12.9 ± 1.2**	**20.2 ± 3.7**	**0.034**
Interleukin-18	**40.1 ± 5.1**	**60.9 ± 8.8**	**0.029**
IP-10	153.0 ± 30.4	137.1 ± 39.8	0.377
LIX/CXCL5	ND	68.3* (1)	NC
Leptin	86.5 ± 18.3	110.0 ± 17.9	0.191
MCP-1	143.0 ± 30.6	187.2 ± 33.5	0.175
MIP-1 alpha	ND	ND	NC
MIP-2	93.6 ± 6.5	110.2 ± 11.2	0.106
RANTES/CCL5	33.3 ± 5.5	32.6 ± 6.8	0.466
TNF alpha	0.6 ± 0.2* (2)	1.3 ± 0.9* (2)	NC
VEGF	14.1 ± 1.6	18.3 ± 2.2	0.071

* Denotes mean ± SEM of incomplete datasets due to some samples falling below detectable range. Bold text represent statistically significant differences between the means (*P* < 0.05). The numbers in parentheses represent the number of samples with detectable levels. ND, none detected; NC, not calculated; EGF, epidermal growth factor; G-CSF, granulocyte colony-stimulating factor; GM-CSF, granulocyte macrophage colony-stimulating factor; GRO/KC/CINC, Growth-regulated oncogene/keratinocyte chemoattractant/cytokine-induced neutrophil chemoattractant; IP-10, Interferon gamma-induced protein 10; LIX, Lipopolysaccharide-induced CXC chemokine; MCP-1, monocyte chemotactic protein 1; MIP, macrophage inflammatory protein; RANTES, regulated on activation, normal T cell expressed and secreted; TNF, tumor necrosis factor; VEGF, vascular endothelial growth factor.

### Placental ischemia and PTZ increases brain water content

**Figure 2:**
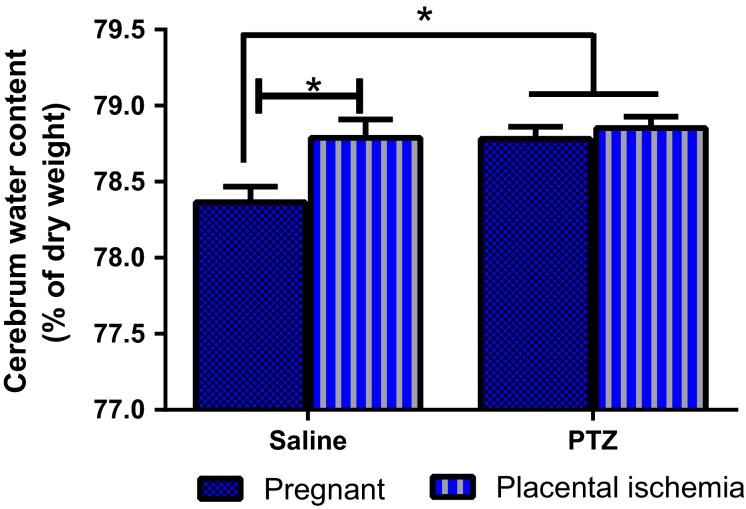
Changes in brain water content in response to placental ischemia and PTZ injection. Placental ischemia and PTZ treatment increases brain water content in pregnant rats. Bars represent Mean ± SEM. *P < 0.05. *N* = 13–15 per group/treatment.

Brain water content increased significantly in response to placental ischemia (Fig.2, *P* = 0.012) as well as PTZ treatment (*P* = 0.014). Placental ischemia increased brain water content from 78.4 ± 0.1% in the pregnant group to 78.8 ± 0.1% (*P* < 0.05). PTZ increased brain water content in the pregnant group to 78.8 ± 0.1% (*P* < 0.05) and did not change the brain water content in the placental ischemic group (78.9 ± 0.1%).

### PTZ increases circulating neurokinin-B levels

**Figure 3:**
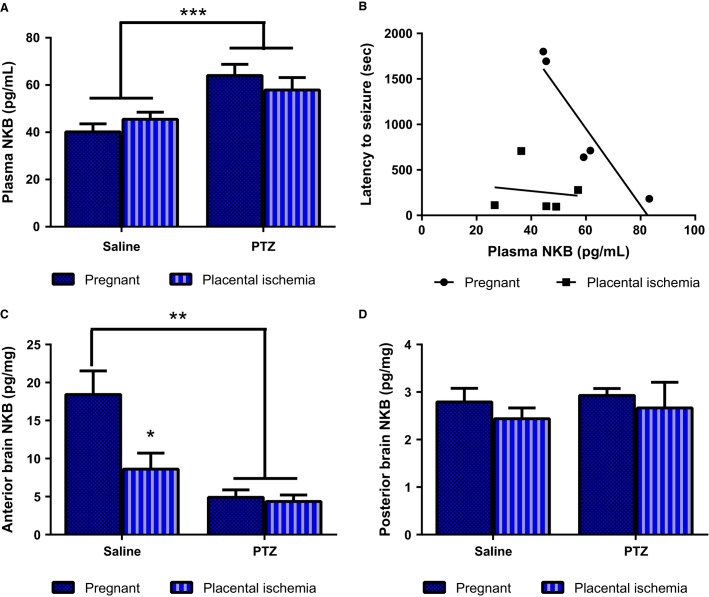
Changes in plasma and brain levels of neurokinin B. (A) Plasma NKB levels are increased in response to PTZ treatment. (B) Relationship between plasma NKB levels and latency to seizures. (C) Anterior brain NKB levels. (D) Posterior brain NKB levels. Bars represent Mean ± SEM. ***P < 0.001, **P < 0.01, *P < 0.05. *N* = 11–13 per group/treatment.

There was a main effect of PTZ treatment on circulating neurokinin-B levels (Fig.3A, *P* < 0.001). Placental ischemia had no effect on plasma neurokinin-B levels (40.2 ± 3.4 pg/mL in pregnant untreated rats vs. 45.5 ± 3.0 pg/mL in untreated placental ischemic rats). PTZ increased neurokinin B levels in the pregnant rat to 64.0 ± 4.7 pg/mL (*P* < 0.05) and placental ischemic rats to 57.9 ± 5.3 pg/mL (*P* < 0.05). The level of neurokinin B present in the circulation was negatively correlated to the latency to seizures in the pregnant group (slope = −42.19; *R*^2^ = 0.877; *P* = 0.019); however, there was no correlation between the circulating neurokinin B levels and latency to seizure in the placental ischemic group (slope = −3.06; *R*^2^ = 0.019; *P* = 0.826).

Neither placental ischemia nor PTZ had an effect on the expression of neurokinin B protein levels in the placenta (data not shown). However, placental ischemia induced a significant reduction in neurokinin B in the anterior cerebrum (*P* = 0.034; Fig.3C) but no change in the posterior cerebrum (*P* > 0.05; Fig.3D). PTZ treatment led to a reduction in neurokinin B expression in both the normal pregnant and placental ischemic rats in the anterior cerebrum (*P* = 0.001), but had no effect in the posterior cerebrum (*P* > 0.05).

## Discussion

Eclampsia is diagnosed when the manifestation of seizures or unexplained coma occurs during pregnancy or the postpartum period in a pregnant or preeclamptic woman with no previously diagnosed seizure disorder. The mechanisms contributing to seizures in eclampsia patients are not known. This study presents the rat model of placental ischemia combined with PTZ treatment as a potential model of eclampsia. Results indicate that placental ischemia led to a reduced latency or hastened onset of first seizure in response to a subconvulsive dose of PTZ.

While it is well established that preeclampsia and the rat model of placental ischemia are characterized by increased levels of circulating and placental inflammatory cytokines, it is not known whether cerebrospinal fluid levels of cytokines/chemokines are altered. Because the cerebrospinal fluid has direct contact with the brain and surface vessels, increased cytokines in the cerebrospinal fluid can directly affect brain signaling and vascular function. In the current study, increased cerebrospinal fluid levels of eotaxin, IL-2, IL-17, and IL-18 were observed in the placental ischemic rat. Eotaxin (CCL11) is a chemoattractant for eosinophils and basophils. In the central nervous system, eotaxin is released by activated astrocytes and act on microglia to promote glutamate-induced neurotoxicity (Parajuli et al. 2015). Therefore, the increase in CSF eotaxin level in response to placental ischemia suggests potential increased glutamate activity and neurotoxicity. Future studies will determine whether placental ischemia is associated with increased glutamate signaling. A review of the literature revealed no studies reporting changes in eotaxin levels during preeclampsia. However, increased circulating (plasma and serum) levels of IL-2 (Szarka et al. 2010; Molvarec et al. 2011), IL-17 (Martínez-García et al. 2011; Toldi et al. 2011; Darmochwal-Kolarz et al. 2012), and IL-18 (Huang et al. 2005; Seol et al. 2009; El-Kabarity and Naguib 2011) have been shown in preeclamptic patients compared to normotensive pregnant controls. Additionally, infusion of IL-17 into the pregnant rat increases mean arterial pressure and oxidative stress (Dhillion et al. 2012) while reducing IL-17 using the soluble receptor to IL-17 into placental ischemic rats attenuates blood pressure and oxidative stress (Cornelius et al. 2013). Moreover, the lymphatic system has been described as a system for the collection of products from the extracellular space into the cerebrospinal fluid (Iliff and Nedergaard 2013; Mendelsohn and Larrick 2013; Plog et al. 2015). Thus, the increases in cerebrospinal fluid cytokines could indicate increased inflammation at the tissue level, potentially through increased production by neuroglia.

Eclamptic patients have been reported to present with various cerebrovascular abnormalities, including cerebral edema. Therefore, this study assessed the brain water content in rats subjected to placental ischemia with or without PTZ treatment. Results indicate that brain water content increased in response to placental ischemia as shown previously (Warrington et al. 2014), but was also increased with PTZ treatment. While there was only a small increase in brain water content in response to placental ischemia and PTZ treatment, the brain sits in the enclosed space of the skull and has limited space to expand. Additionally, this increase in brain water content is consistent with findings from previous studies (Amburgey et al. 2010; Ryan et al. 2011; Warrington et al. 2014, 2015). Additionally, cerebral edema has been reported in preeclamptic patients (Mitas and Rogulski 2012), eclamptic patients (Matsuda et al. 2005; Takeuchi et al. 2005; Hirashima et al. 2006), epileptic patients (Ali et al. 2013), and the placental ischemic model (Ryan et al. 2011; Warrington et al. 2014). Based on the finding that both placental ischemia and PTZ injection increased brain water content, it is possible that seizure activity can induce edema and the presence of edema itself may contribute to increased seizure susceptibility.

Neurokinin B (NKB) is a neuropeptide belonging to the family of tachykinins that also includes substance P and neurokinin A. NKB has been shown to be increased in the placenta and circulation of preeclamptic women (Page et al. 2000; D’Anna et al. 2004; Liu et al. 2009). NKB is increased in the rat hippocampus following PTZ or kainic acid- induced seizures (Marksteiner et al. 1992) and its receptor, NK3, increases in the cerebellum following kainic acid-induced seizures (Röder et al. 1994). In this current study, NKB levels were reduced in the anterior cerebrum of rats subjected to PTZ. This difference in results could be a result of the following: (1) the current study utilized a single injection of PTZ while other studies use kindling methods that require two or more injections of the pro-convulsive substance; (2) the current study assessed acute changes (30 min following seizure initiation) while other studies measured changes 24 h to months following seizure initiation. It is very interesting that placental ischemia by itself led to a decrease in NKB expression in the anterior brain, the same region where previous study demonstrated increased edema and blood-brain barrier permeability (Warrington et al. 2014). It is possible that reductions in NKB expression could be compensatory as NKB has been described as a proconvulsive neuropeptide (Maubach et al. 1998). NKB concentration was significantly increased in the circulation following PTZ injection but placental expression was unchanged. Thus, placental ischemia is not associated with increased production of NKB from the placenta. Because the decrease in NKB expression in the brain was associated with an increase in circulating levels of NKB, it is possible that NKB spillover into the circulation occurred in response to PTZ treatment.

Increased neurokinin B in the circulation could serve to increase blood-brain barrier permeability and contribute to cerebral edema formation. Indeed, neurokinin B has been shown to cause vasorelaxation in cerebral vessels in humans and various animal models (Jansen et al. 1991). Additionally both neurokinin B and its receptor, NK1 have been shown to increase plasma extravasation in the lung and ear (Inoue et al. 1996; Grant et al. 2002a, b). Taken together, PTZ may induce edema through increased circulating NKB levels.

In conclusion, the placental ischemic model presents an excellent animal model for studying mechanisms for increased seizure susceptibility. While there are limitations to this model in that the seizures are not spontaneous but are drug-induced and may not directly mimic what occurs in eclamptic patients, this animal model shares numerous characteristics to the clinical condition of preeclampsia. Thus, by targeting specific pathways altered in preeclampsia, insights can be drawn into specific pathways that may trigger seizure induction and studies can be designed to elucidate whether blocking some of these pathways can improve seizure severity or prevent seizures altogether.
